# Supplementary material for: CsPb(Br/Cl)3 Perovskite Nanocrystals with Bright Blue Emission Synergistically Modified by Calcium Halide and Ammonium Ion
Source: Nanomaterials (Basel). 2022 Jun 13;12(12):2026. doi: 10.3390/nano12122026 (PMC9231175; doi:10.3390/nano12122026)
Supplement: Supplementary file 1 [file nanomaterials-12-02026-s001.zip › nanomaterials-1733683-supplementary.pdf]

Supporting Information

$CsPb(Br/Cl)_3$ Perovskite Nanocrystals with Bright Blue Emission Synergistically Modified by Calcium Halide and Ammonium Ion

Weizhuo Zhang, Xin Li, Chencheng Peng, Fei Yang, Linyuan Lian, Runda Guo, Jianbing Zhang * and Lei Wang *

Wuhan National Laboratory for Optoelectronics, School of Optical and Electronic Information, Huazhong University of Science and Technology, Wuhan 430074, China; 924823606@qq.com (W.Z.); lixin19992017@163.com (X.L.); pengchenchengupup@163.com (C.P.); 18390241891@163.com (F.Y.); 381318624@qq.com (L.L.); runda_guo@hust.edu.cn (R.G.)

* Correspondence: jbzhang@hust.edu.cn (J.Z.); wanglei@mail.hust.edu.cn (L.W.)

Characterization

The PL spectra and PLQY of $CsPb(Br/Cl)_3$ NCs were measured through Hamamatsu Quantaurus-QY C11347-11 with an excitation peak of 405 nm (The NCs solution diluted with hexane is placed in a cuvette, placed in an integrating sphere system). Time-Resolved PL decay was detected by Time-Correlated Single-Photo Counting by Edinburgh FLS920 with a 375 nm laser. TEM and HRTEM images and Energy-Dispersive X-ray Spectroscopy were obtained utilizing Tecnai G2 20 U-Twin high-resolution scanning TEM operated at an acceleration voltage of 200 kV. The UV-Vis Absorption spectra of these $CsPb(Br/Cl)_3$ NCs were measured with Shimadzu UV-3600 ultraviolet-visible-near-infrared (UV-vis-NIR) spectrophotometer. X-ray Diffraction(XRD) profiles of these $CsPb(Br/Cl)_3$ NC films were performed using PANalytical PW3040-60 materials research X-ray diffractometer. Fourier Transform Infrared Spectroscopy (FTIR) of these powder samples was conducted with tablet synthesizing by 1 mg PNCs powder mixing with 100 mg KBr. X-ray Photoelectron Spectroscopy(XPS) spectra of $CsPb(Br/Cl)_3$ NCs deposited on a silicon wafer were collected on Kratos AXIS-ULTRA DLD-600W. The device's PL spectra, luminescence, and power efficiency were measured by Keithley 2400 source measurement unit and PhotoResearch spectroradiometer (PR655).

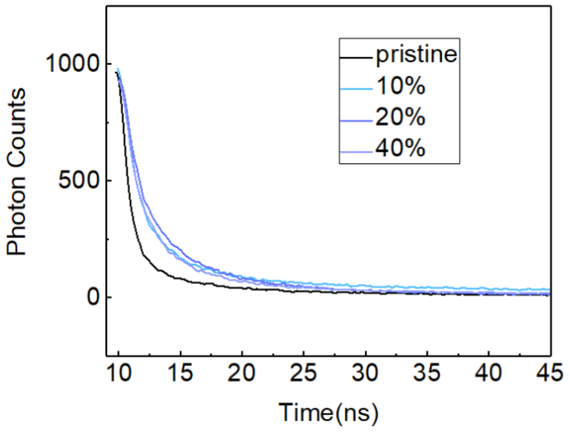

Figure S1. PL decay curve for samples with the different feed ratios of ammonium to cesium in synthesis, no calcium in all samples. The feed ratio are 0 (for pristine), 10%, 20% and 40%, respectively.

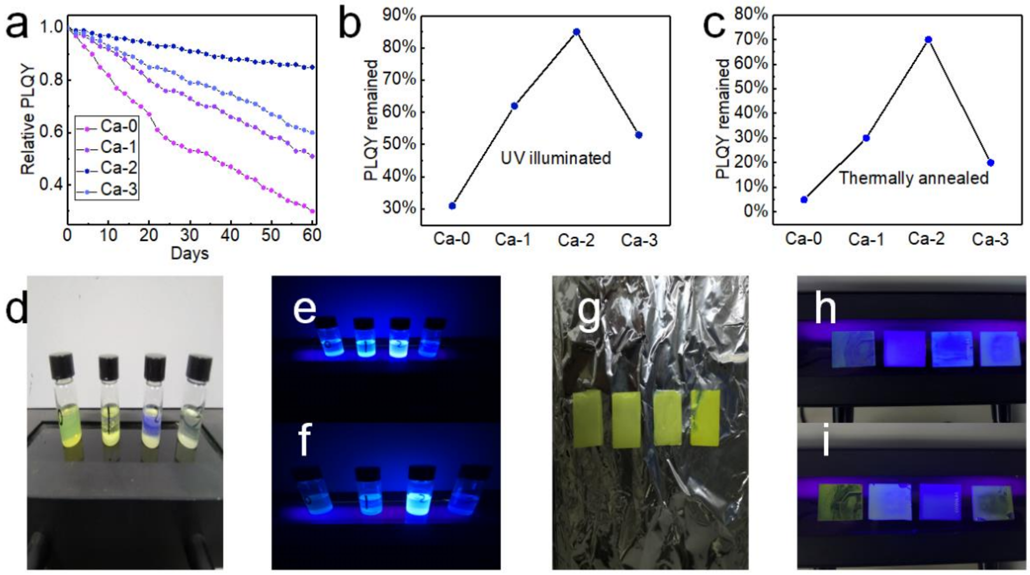

Figure S2. (**a**) PLQY record for Ca-*i* samples varied with different days, showing stability under atmospheric conditions. (**b**) PLQY remained after continuous UV irradiation for each sample. (**c**) PLQY remained after thermal treatment for Ca-*i* samples. (**d**) Pictures for Ca-*i* samples after UV irradiation shot without UV excitation. (**e**) Pictures for Ca-*i* samples at the beginning of the UV irradiation stability test. (**f**) Pictures for Ca-*i* samples after continuous UV irradiation. (**g**) Images for Ca-*i* deposited films on glass ready to be thermally treated on the hot plate with tin paper. (**h**) Luminescence of each sample deposited film before thermal treatment. (**i**) Luminescence of each sample deposited film after thermal treatment, shot after moving from hot plate to UV light.

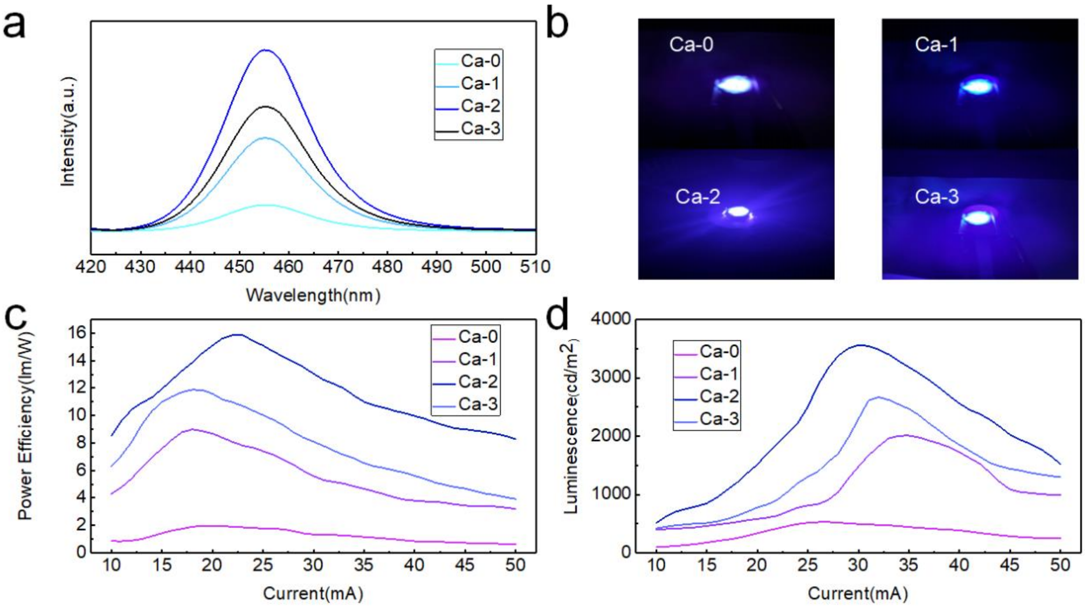

Figure S3. (**a**) PL spectra of the blue device fabricated by our deep blue perovskite NCs. (**b**) Images for blue devices based on Ca-*i* samples. (**c**) Power efficiency for each device. (**d**) Luminescence for each device.

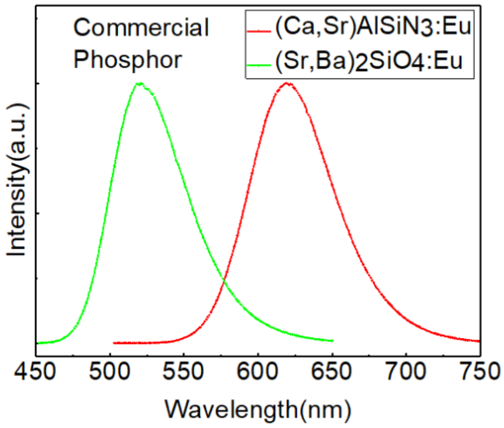

Figure S4. PL spectra of two commercial phosphors utilized in the fabrication of white devices.

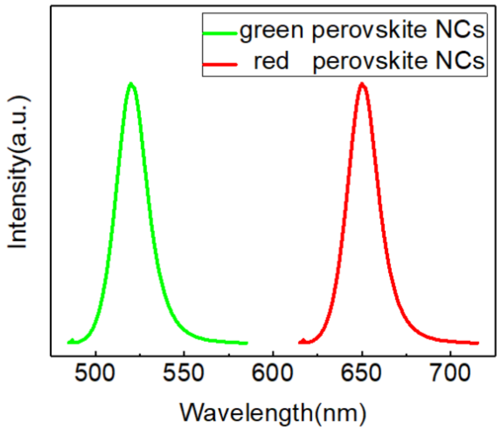

Figure S5. Perovskite NCs emitting green and red light synthesized with a similar modification process.

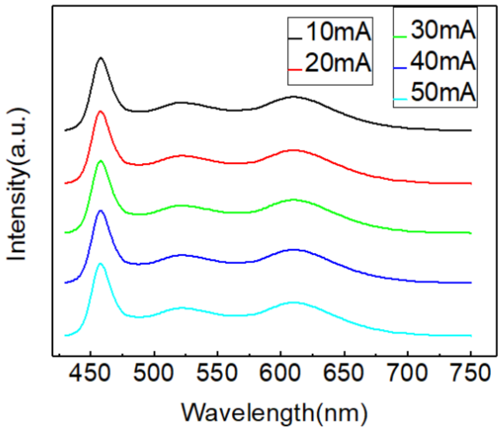

Figure S6. PL spectra of the white device under different operating currents.

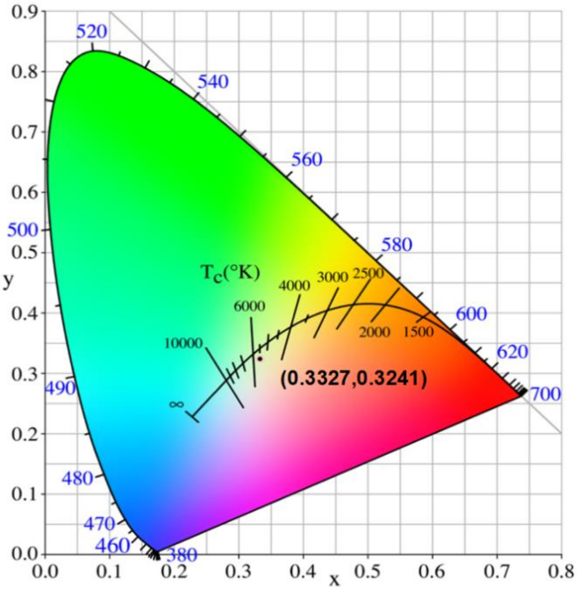

Figure S7. Coordinate of the white device, which is very close to standard white emission (0.33, 0.33).

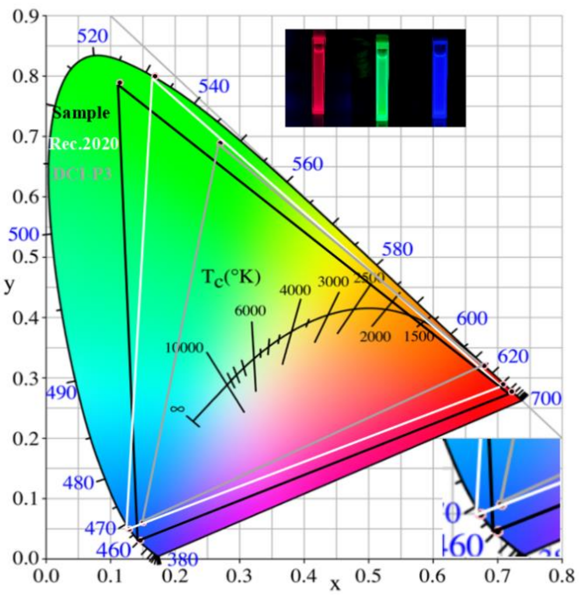

Figure S8. Color gamut comparison of our three primary color perovskite NCs, commercial DCI-P3 standard, and most advanced Rec.2020 standard.

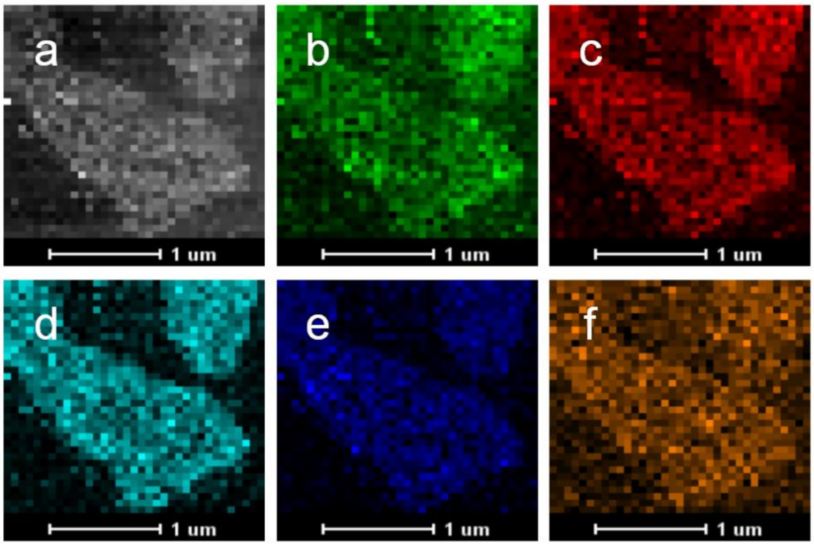

Figure S9. Energy-dispersive x-ray spectroscopy images for different elements in NCs. (**a**) Image of NCs. (**b**–**e**) Images represent Br, Cl, Cs, Pb, and Ca, respectively.

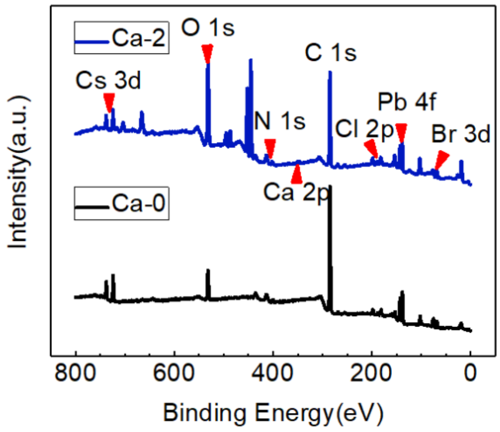

Figure S10. XPS spectra of all element peaks for Ca-0 and Ca-2 samples.

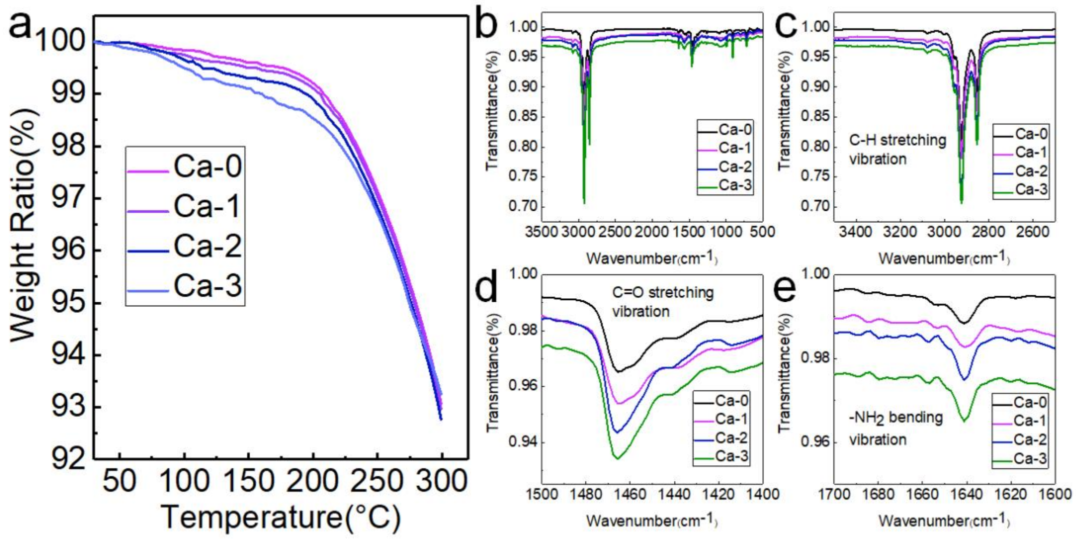

Figure S11. (**a**) Thermal gravity analysis results for Ca-*i* samples. (**b**) FTIR spectra for each sample. (**c**) C–H stretching vibration spectra. (**d**) C=O stretching vibration spectra. (**e**) –NH_2 bending vibration spectra.

Table S1. PL decay parameters for Ca-*i* samples.

**Sample**	**PLQY (%)**	**A_1**	**t_1 (ns)**	**A_2**	**t_2 (ns)**	**t_{avg} (ns)**
Ca-0	13%	0.9986	1.499	0.0014	6.849	1.533
Ca-1	49%	0.9878	2.192	0.0122	10.316	2.638
Ca-2	93%	0.7392	5.085	0.2608	14.772	9.989
Ca-3	63%	0.9059	3.976	0.0941	11.183	5.606

Table. S2 TA parameters for Ca-0 and Ca-2 samples.

**Sample**	**PLQY (%)**	**P_1**	**t_1 (ps)**	**P_2**	**t_2 (ps)**	**P_3**	**t_3 (ps)**
Ca-0	13%	0.463	9.597	0.333	75.362	0.204	2219.595
Ca-2	93%	0.318	6.206	0.114	70.439	0.568	2991.312
